# Analysis of factors influencing the results of colorectal cancer surgery in the elderly

**DOI:** 10.1186/1471-2482-13-S1-A44

**Published:** 2013-09-16

**Authors:** M Saracco, A Esposito, DG Palmieri, N Carlomagno, A Renda

**Affiliations:** 1 Chirurgia Generale ad Indirizzo Addominale, Università Federico II, via Pansini 5 Napoli, Italy

## Introduction

Since over 70% of colorectal cancers (CRC) occurs in patients at least aged 65 years old, it can be considered a disease of elderly people and is one of major cause of morbidity and mortality at this age [1][2][3]. The purpose of this study is to evaluate the outcome of surgical treatment in older population referred to our institution and the effectiveness in terms of surgical and oncologic results with emphasis to age-related risk factors.

## Methods

A retrospective analysis of a geriatric population (>70 y.o.) affected by CRC was carried out to review risks and benefits of surgery. In our series of 120 patients treated during the period 2008–2012 69 patients (57,5%) were at least 70 year old (median age 74 years) and 12 of these (17,4%) patients were octogenarians. 37 were male and 32 female. The following parameters have been evaluated: presence of co-morbidities, adoption of neoadjuvant therapies, pathology, surgical treatment and we tried to understand their feasible role to determine results in terms of morbidity, mortality, disease free survival and 2yr-survival 4.

## Results

Co-morbidities age-related, such as diabetes mellitus, cardiopulmonary disease, and chronic renal disease, were present in 52 (75%) patients. Combined neoadjuvant therapies based on radio-chemotherapy protocols (RT-CHT) were carried successfully out in 5 patients with mid and low rectal cancer. Our surgical choices are synthesized in table 1.

**Table 1:** CRC in elderly patients: our surgical choices according to the site of tumors

Left-sides tumors (62,3%) 43	Right-sides tumors (34,8%) 24
Anterior resection 31	Right colectomy 24

Left colectomy 4	

Hartmann’s procedure 2	

Miles’ procedure 1	

Total colectomy with ileo rectal anastomosis 5	

Surgery was performed in emergency in 2/69 (2,3%) cases. A simple stoma was done in 2 cases. In 7 patients synchronous hepatic metastasis were diagnosed and 3 cases presented a peritoneal carcinosis.

The majority of tumors presented type I cell grading (G1). Tumor markers were not significant in 47 cases (66%). Tumors resulted at the following TNM’s stages: T_1_N_0_M_0_ 3%, T_2_N_0_M_0_ 3,5%, T_3_N_0_M_0_ 25%, T_2_N_1_M_0_ 7%, T_3/4_N_1/2_M_0_ 47% and T_N_N_N_M_1_ 14,5%.

In elderly people post- operative complications occurred in 20 cases (29%) and 13% concerned medical complications. Perioperative mortality rate was 0,5%. Mean hospitalization was 15 days (range 12-18 days). After 24 months the overall survival was 81.4% for patients >70 years and the disease-free survival was 73,9%.

## Conclusions

Recent studies showed that elderly patients do not have to receive sub-optimal surgical and medical treatments because of unacceptable prejudices. The relationship between age and outcomes is complex and depends on differences in tumor-stage and pre-existing co-morbidities [5]. Chronological age alone does not provide sufficient guidance for surgical effectiveness. Key parameters in the surgical risk assessment are represented by biological indexes of each patient. The major complications were analyzed in according to ASA score that, strongly depending on clinical functional status, was a valid preoperative parameter able to predict the surgical morbidity. The oncologic results and the morbidity rates of our experience confirm that surgery is a safe therapy also in elderly patients when operative risk and associated diseases evaluation has been carefully calculated. Our morbidity and mortality rates are acceptable. Our results do not differ from those reported in literature for younger patients and from other series concerning elderly population. In term of prognosis the analysis of overall survival and disease-free survival at 24 months showed a better trend in a geriatric cohort of patients with age >70 years. The role of surgery in colorectal cancer geriatric patients is well defined in the scientific literature. More comprehensive insights in oncologic surgery of elderly population will derive from the better understanding of the physiology of aging and combined therapies.
